# Can adaptive hyperactivation result in a positive score on the Mood Disorder Questionnaire? Evidence from a case-control study over a community survey

**DOI:** 10.3389/fpsyt.2025.1626277

**Published:** 2026-01-05

**Authors:** Michela Atzeni, Michele Fornaro, Massimo Tusconi, Cesar Ivan Aviles Gonzalez, Elisa Cantone, Elisa Pintus, Serdar M. Dursun, Antonio E. Nardi, Federica Sancassiani, Mauro Giovanni Carta

**Affiliations:** 1 Department of Medical Sciences and Public Health, University of Cagliari, Cagliari, Italy; 2 Department of Neuroscience, Reproductive Science and Odontostomatology, Federico II University of Naples, Naples, Italy; 3 Center for Liaison Psychiatry and Psychosomatics, University Hospital of Cagliari, Cagliari, Italy; 4 Department of Medicine and Surgery, University of Kore, Enna, EN, Italy; 5 Neurochemical Research Unit, Department of Psychiatry, University of Alberta, Edmonton, AB, Canada; 6 Instituto de Psiquiatria, Universidade Federal do Rio de Janeiro, Rio de Janeiro, RJ, Brazil; 7 PhD Program in Tropical Medicine, Universidad Popular del Cesar, Valledupar, Colombia; 8 Department of Nursing, Universidad Popular del Cesar, Valledupar, Colombia

**Keywords:** BD, bipolar disorders, early diagnosis, hyperactivation, hyperactive, MDQ, mood disorder, prevention

## Abstract

**Background:**

Bipolar Disorder (BD) remains challenging to identify, and the Mood Disorder Questionnaire (MDQ) may capture heterogeneous forms of hyperactivation, including adaptive patterns unrelated to psychopathology.

**Objectives:**

To determine whether MDQ-positive individuals include a subgroup with adaptive hyperactivation—characterized by high quality of life (QoL) and minimal psychiatric morbidity—and to examine whether MDQ positivity also identifies diagnoses beyond BD.

**Methods:**

Using data from a large Italian community survey with DSM-IV clinical interviews and MDQ screening, we conducted a case–control analysis. MDQ-positive individuals were stratified by SF-12 QoL scores (>40 vs. ≤40), and matched MDQ-negative controls were selected by age and sex. Psychiatric diagnoses were compared using ANOVA and chi-square tests.

**Results:**

Among 91 MDQ-positive participants, 33% showed high QoL and exhibited markedly fewer psychiatric diagnoses than those with low QoL (χ²=15.529, p<0.0001). High-QoL MDQ-positive individuals displayed psychiatric morbidity comparable to MDQ-negative controls, whereas low-QoL MDQ-positive individuals showed excess anxiety, obsessive–compulsive, and eating disorders.

**Conclusions:**

MDQ positivity identifies a heterogeneous population, including individuals with adaptive hyperactivation and preserved functioning. These findings highlight the need for more refined instruments capable of distinguishing adaptive from pathological hyperactivation and caution against over-pathologizing MDQ positivity in clinical and public health settings.

## Introduction

Some decades ago, there was a strong interest in developing paper and pencil screeners for bipolar disorders due to the delay in diagnosing and the difficulty in detecting episodes of previous hypomania among people with major depressive episodes (1). The Mood Disorder Questionnaire (MDQ) (2–5) was the most employed tool in both clinical and, especially, epidemiological surveys. Community survey adopting MDQ found a lifetime prevalence of around 4% in the US (3), 3.6% in France (6), 3% in Italy (7), and 4.3% in South Korea (8); frequencies higher than those, under 2%, found in surveys conducted using interviews (9).

However, a series of studies indicated that MDQ produced false positives concerning a gold standard of a diagnosis of Bipolar Disorder (BD) conducted according to the criteria of the international classifications (10–13). The “false MDQ positives” received diagnoses of post-traumatic stress disorder, borderline personality disorders, anxiety disorders, attention deficit disorder, eating disorders, alcohol and drug use disorders, and impulse control disorder (10–13). Therefore, the episodes of hyper-energy/hyper-activity detected by MDQ did not coincide precisely with mania or hypomania typical of bipolar disorders. It was underlined that even if the “positives” did not fulfill the whole criteria for BD, they show several homogeneities with BD (1) regarding sex and age distributions, the high level of distress, the low social functioning, and the average low perception of quality of life (14–16). Even the risk of suicide was found elevated in “positives,” according to what happens in people with a diagnosis of BD, specifically in young MDQ positives likely to develop a diagnosis of BD in the future (17–20). In addition, it was noted that all the psychiatric diagnoses found in false MDQ positives had, in several studies, been found to be strictly associated with BD (1); the same comorbid diagnosis was even diagnosed ten years, on average, before the diagnosis of the comorbid BD (21). Comorbidity with bipolar disorders has been documented in post-traumatic stress disorder (22–26), specific phobia (27–29), panic disorder (30–32), attention-deficit hyperactivity disorder (33–35), alcohol use disorders (36–38), substance use disorders (39–41), eating disorders (42–44), impulse control disorder (45–47), and borderline personality disorders (48–50), the official classification of the American Psychiatric Association (51) stated that bipolar disorder was “severe” disorders separated from the depressive ones, even, but not only, according to the evidence of the low prevalence of BD in the community and the weakness of the current of thought which argued an expansion of the area of BD, this point of view was also followed with all the “official psychiatry.” But this concept was contrasted by a minority of “neo-Kraepelinian” psychiatrists, for which the cases of “full” bipolar disorder as diagnosed by the official classifications were interpreted as the emerging tip of an iceberg with a significant amount of “submerged conditions” (most of them classified as depressive disorders) showing subthreshold features, courses and treatment response similar to BD, thus underlined a “bipolar spectrum disorders area” (20, 52–54).

A third view has been advanced more recently. It was underlined that the MDQ positivity was not associated with the presence of some genetic variants strictly linked with bipolar disorder. However, both MDQ and the presence of the same specific genetic variants, if used as screeners, showed some complementary accuracy in identifying bipolar disorders (55, 56). At the same time, some genetic characteristics known as typical of bipolar disorder were found with high frequency in older adults free of bipolar or other psychiatric disorders and with high adjustment levels and traits of hyperactivity/hyper-energy and novelty seeking (57, 58). Furthermore, MDQ positivity was found to have a strict genetic association with other conditions, such as post-traumatic stress disorder, anxiety disorders, and insomnia (59). Insomnia is one of the most relevant elements of the so-called “social rhythm”, whose association with stress and psychopathological relevance has been recently emphasized (60–62). MDQ positivity was found to be associated with the presence of altered social rhythms (63). Another interesting theme concerns the relationship between a positive score on the MDQ and an impaired perception of Health-related Quality of Life. A community survey found that the impairment in health-related quality of life in individuals who scored positive on the screener, regardless of comorbid conditions, even when people with MDQ positives and diagnosis of bipolar disorder and Depressive disorders were excluded (57), or even other diagnoses were excluded (64). This type of association has also been found in older adults who tested positive for the MDQ, and this makes it very unlikely that these are forms that will develop into a full-blown disorder with a psychiatric diagnosis in the future (57, 64). Further evidence also confirmed that a positive score on the screener identifies an area of people without a pathologic psychiatric status but with a low quality of life (57, 64). It should be noted, however, that people with a positive MDQ without other psychiatric diagnoses had a low mean score on the scales of the perception of quality of life but with a significant standard deviation, thus leading to the hypothesis that there were also several people with a high level of quality-of-life positives to the MDQ (57, 64).

According to this evidence was supposed to the existence of three different hyperactivation levels: the first one could be due to an adaptive increase in energy/activation as shown in athletes of excellence before and/or after a high performance (65); the second level, it is an activation in the association of dysregulation of social and biological rhythms due to continuous stimulation of stress hormones, with a potential positive score at MDQ but without reaching the threshold for a diagnosis of mania or hypomania, like in burnout syndromes at work and called Dysregulation of Mood, Energy, and Social Rhythms Syndrome (DYMERS) (57). DYMERS may evolve in other disorders (even but not only BD) in balance with the specific/personality susceptibility and the nature/level of the stressor (66, 67). The third level of hyperactivity detected by MDQ is that of full manic or hypomanic episodes.

The main objective of the present work is to confirm this hypothesis and, specifically, to verify if, among the positives screened by the MDQ, it is possible to identify a group of people with excellent adjustment, thus with an “adaptive hyperactivation.” A secondary objective of this study is to verify if MDQ can screen for diagnoses other than bipolar disorders.

In the context of this study, *adaptive hyperactivation* refers to transient or trait-like increases in energy, motivation, and activity levels that enhance rather than impair psychosocial functioning. Conceptually, it describes a pattern of heightened activation associated with high quality of life, preserved social and occupational adjustment, and absence of significant psychiatric morbidity. Operationally, in the present analysis, adaptive hyperactivation was inferred in MDQ-positive individuals who scored above 40 on the SF-12 and exhibited no excess of DSM-IV psychiatric diagnoses compared with matched MDQ-negative controls. This construct is distinguished from dysregulated activation states—such as those related to mood disorders or rhythm disruption—by its functional and non-pathological nature.

The study uses a database of a community survey that adopted both structured diagnoses according to the DSM-IV (conducted by clinicians) and the MDQ screening questionnaire.

## Methods

Design of the study: Case-control study from the database of a community survey.

Recruitment Methods and Study Sample: The database of the research comes from interviews with people randomly selected by stratification by cells (heights cells by age and sex) from municipality records (>17 years old) of urban and rural municipalities in six different Italian regions, A detailed and extended description of the methodology of the community survey including sampling methods, interviews, and data analysis has been already published (7, 68).

The original community survey approached 2,250 individuals, of whom 1,921 (85.3%) agreed to participate and completed the face-to-face assessment. Among them, 1,853 participants (96.5%) provided complete data for both the MDQ and the ANTAS clinical interview. The present analyses excluded individuals with missing MDQ responses (n = 41) or incomplete SF-12 data (n = 27), as well as those whose diagnostic interviews could not be fully validated due to recording or transcription inconsistencies (n = 14). The final analytic sample therefore comprised 1,771 participants (78.7% of those initially contacted). From this cohort, 91 individuals met criteria for MDQ positivity and were included as cases in the present study, alongside their matched controls.

### Control selection and matching procedure

To ensure comparability between groups, MDQ-negative controls were selected through a structured matching procedure. For each MDQ-positive participant, we generated a matching cell defined by sex and 5-year age band, using the complete pool of MDQ-negative individuals with valid ANTAS and SF-12 data. Within each cell, we identified all eligible controls and applied random sampling without replacement. For MDQ-positive individuals with SF-12 ≤40 (low QoL), two matched controls were drawn from the corresponding cell (2:1 ratio). For MDQ-positive individuals with SF-12 >40 (high QoL), a 1:1 matching ratio was used because of the larger number of eligible cases in this subgroup. Matching was exact on sex and age band, and all selected controls were excluded from subsequent sampling to avoid duplication.

Participants with missing MDQ responses, incomplete SF-12 scores, or non-valid ANTAS interviews were excluded prior to the matching process. This procedure ensured consistent demographic alignment and allowed unbiased comparison of psychiatric morbidity between cases and controls.

The group of cases consists of all the people positive on the MDQ using a low threshold, i.e., only based on scoring positivity to at least 7 of the first 13 items. The group of cases was divided based on whether or not they obtained a score on the SF-12 equal to or greater than 40, which indicates a high perception of quality of life.

For each MDQ-positive case with SF-12 ≤40, a cell was created with all the people eligible to be matched as a control for that case. Then, we randomly selected two controls for each case. A similar method was used for Cases with SF-12 >40, but for this group, we randomized only two controls for each case due to the high number of cases. We then verified whether the frequency of specific psychiatric diagnoses and the total number of diagnoses in people who tested positive for the MDQ but had high satisfaction with their quality of life was like the frequencies in people who tested positive for the MDQ but had a low quality of life and in people with a high quality of life and negative at MDQ.

### Brief description of the original survey methodology

The community survey employed a multistage, stratified sampling strategy based on municipal registries, ensuring representativeness across age, sex, and urban-rural residence. Eligible participants were contacted by trained clinicians and invited to take part in face-to-face assessments conducted in their homes or in designated municipal facilities. All interviews were performed by medical doctors or clinical psychologists who underwent standardized training and inter-rater reliability calibration prior to fieldwork.

The diagnostic assessment followed a two-step structure. First, socio-demographic characteristics and health-related information were collected through an *ad hoc* questionnaire. Second, lifetime DSM-IV psychiatric diagnoses were established using the Advanced Neuropsychiatric Tools and Assessment Schedule (ANTAS), a validated semi-structured interview. Measures of quality of life were obtained through the SF-12, while lifetime hypomanic/activation symptoms were screened using the Italian version of the Mood Disorder Questionnaire (MDQ). The survey protocol incorporated rigorous quality-control procedures, including periodic supervision, on-site auditing, and consistency checks across interviewers.

### Study tools

Demographic data were assessed using an *ad hoc* instrument. A medical doctor or clinical psychologist conducted the DSM-IV psychiatric diagnosis during the community survey utilizing the Advanced Neuropsychiatric Tools and Assessment Schedule (ANTAS), a validated semi-structured clinical interview (69). The lifetime hypomanic/activation episodes were identified using the Italian version of the Mood Disorder Questionnaire (MDQ) (4, 70). The Quality of Life (QoL) perception was measured using the score at the Health Survey Short Form (SF-12). The SF-12 tool gives information on several dimensions of QoL, such as physical and emotional state, general health, vitality, and pain (71).

### SF-12 threshold

In line with prior epidemiological studies conducted in Italian and European community samples, an SF-12 score greater than 40 was used to indicate high perceived quality of life. This threshold reflects the point above which individuals typically fall within the normative or above-norm functional range across physical and mental health domains. Previous research examining MDQ positivity and health-related functioning within similar populations has adopted the same criterion, supporting its validity for distinguishing individuals with preserved adjustment from those with impaired well-being.

### Assessment of intellectual disability

Within the original community survey, the ANTAS interview incorporated standardized items evaluating global cognitive functioning and developmental history, allowing clinicians to identify features consistent with DSM-IV intellectual disability. Participants with evident cognitive impairment or with limitations preventing a reliable clinical interview were excluded during fieldwork. No individuals meeting criteria for intellectual disability were included in the final analytic sample.

### Ethics

The protocol for the Italian community survey, approved by the ethical committee of the Italian National Health Institute (Istituto Superiore di Sanità) (Rome, Italy) and AIFA Grant (Agenzia Italiana del Farmaco, Italian Drug Agency), Number FARM54S73S, started on 1 August 2006; the approval involved verifying the validity of the instruments used based on the results of the research sample. Each interviewee signed informed consent before the interview. This study was conducted by the ethical standards of the Declaration of Helsinki and approved by the local ethics committees in each participating region. All participants obtained Written informed consent before being included in the survey.

### Statistical analysis

The comparison of means and standard deviations of the SF-12 scores between cases (MDQ+) and matched controls (MDQ−) was conducted using statistical parametric analyses (ANOVA). The comparison of the number of psychiatric diagnoses between cases and controls was conducted using the Chi-squared test with Yates correction, which is needed for the Fisher exact test. Miettinen’s simplified method calculated odds, ratios, and 95% confidence intervals (72). Statistical significance for nominal data was calculated using the χ2 test and by one-way ANOVA for numerical data.

### Considerations on sample size and statistical power

This study is based on a fixed-size community survey, and therefore an *a priori* power calculation was not applicable. However, the available sample of 91 MDQ-positive individuals is consistent with previous epidemiological case–control analyses using MDQ-based screening. Given the observed distribution of diagnoses, the study retained adequate power (>80%) to detect medium-to-large differences in psychiatric morbidity between high- and low-QoL subgroups (corresponding to effect sizes of OR ≥ 2.5–3.0). Smaller differences, particularly in low-prevalence conditions such as eating disorders or OCD, may remain undetected, and this limitation has been acknowledged. Nonetheless, the significant associations identified for overall psychiatric comorbidity and specific diagnostic categories suggest that the sample size was sufficient to support the main conclusions of the study.

## Results

With the described methodology, 91 positives to MDQ+ were selected, of which 30 (33.0%) had a score of SF-12 equal to or higher than 40. The characteristics of the experimental group, divided into MDQ+ and MDQ-, and of the two relative control groups are reported in **Table 1**. The two experimental subgroups did not differ from each other in terms of sex distribution or average age. Thanks to the matching methodology adopted, the two control groups were perfectly balanced for these variables with the respective experimental groups.

**Table 1** illustrates how in the experimental group of individuals who tested positive for the MDQ but with a high perception of quality of life (which is over 30% of all positives), a higher frequency of all psychiatric diagnoses is found compared to the group of other MDQ positives (with a low perception of quality of life) (chi-square 1df =15.529, p<0.0001); of “other DSM-IV anxiety disorders” (chi-square 1df=4.388, p=0.036). Comparing the same group of people with positive MDQ but with a high perception of quality of life with those who tested negative for the MDQ with a similarly high perception of quality of life, the number of total psychiatric diagnoses is not dissimilar, just as the frequency in specific diagnoses or diagnostic groups such as eating disorders, bipolar disorders, major depressive disorder, post-traumatic stress disorder, obsessive-compulsive disorder, and Other Anxiety DSM-IV disorders is not different. Conversely, comparing positives with a low perception of quality of life with MDQ negatives with a low perception of quality of life, the MDQ positives show a higher frequency of eating disorders (p=0.040, Fisher exact test), obsessive-compulsive disorder (p=0.027, Fisher exact test) and the set of psychiatric diagnoses (chi-square =33.260, p<0.0001).

**Table 1:** Comparison of lifetime prevalence of DSM-IV diagnoses in cases and controls.

Diagnosis	A.HQoL MDQ+ N=30 (%)	B.LQoL MDQ+ N=61	C.HQoL MDQ+ N=60	D.LQoL MDQ- N=61	AvsB Chi-square with Yates (i.n)	AvsC	BvsD
Eating Disorders	1 (3.33)	4 (6.55)	2 (3.33)	0	χ^2^ = 0.001 p=0.999	p=1 Fisher	p=0.040 Fisher
Bipolar Disorder	1 (3.33)	2 (3.38)	0	0	χ^2^ = 0.001 p=0.999	p=0.333 Fisher	p=0.496 Fisher
Major Depressive Disorders	1 (3.33)	7 (11.45)	2 (3.33)	3 (4.92)	χ^2^ = 1.663 p=0.197	p=1 Fisher	p=1.754 P=0.322
PTSD	0	1 (1.64)	0	0	χ^2^ = 0.001 p=0.999	p=1 Fisher	1 Fisher
OCD	0	5 (8.2)	0	0	χ^2^ = 1.263 p=0.261	p=1 Fisher	0.027 Fisher
Other Anxiety DSM-IV Disorders	3 (10)	20 (32.79)	5 (8.33)	5 (8.20)	χ^2^ = 4.388 p=0.036	p=1 Fisher	χ^2^ = 11.320 P<0.0001
Overall Diagnosis	6* (20.0%)	39* (63.9%)	9* (15.0%)	8* (13.1%)	χ^2^ = 15.529 p<0.0001	p=0.402 Fisher	χ^2^ = 33.260 P<0.0001

*Several diagnoses of comorbidity.

When comparing the frequency of Lifetime DSM-IV diagnoses in MDQ positives vs. MDQ negatives (**Table 2**), all diagnoses compared to have a higher frequency in MDQ positives, this difference reaches statistical significance in the comparison between the sum of all diagnoses in the two groups (45 diagnoses out of 91 people vs. 15 out of 121, chi-square 1df=13.143, p<0.0001); in bipolar disorders (3.30% vs. 0%, chi-square 1df=0.046, p=0.044); in obsessive-compulsive disorders (chi-square 1df=6.759, p=0.009).

**Table 2:** Comparison of lifetime prevalence of DSM-IV diagnoses in MDQ positives vs. MDQ negatives.

Diagnosis	MDQ positives N=91 (%)	MDQ negatives N=121 (%)	Chi-square 1df	OR (CI 95%)
Eating Disorders	5 (5.49)	2 (1.65)	χ^2^ = 2.401 p=0.121	3.45 (0.65-18.2)
Bipolar Disorder	3 (3.30)	0 (0)	χ^2^ = 0.046 p=0.044	Inf.
Major Depressive Disorders	8 (8.79)	5 (4.13)	χ^2^ = 1.259 p=0.162	OR = 2.23 (0.7-7.1)
PTSD	1 (1.10)	0 (0)	χ^2^ = 1.325 p=0.250	Inf.
OCD	5 (5.49)	0 0)	χ^2^ = 6.759 p=0.009	Inf.
Other Anxiety DSM-IV Disorders Including (PD = 8)	23 (25.27)	10 (6.21)	χ^2^ = 11.435 p=0.001	OR = 3.75 (1.7-8.3)
Overall Diagnosis	45*	15*	χ^2^ = 13.143 p<0.000	6.91 (3.5-16.63)

*Several diagnoses of comorbidity.

To complement the p-values reported in the tables, we note that the overall psychiatric morbidity was markedly higher among MDQ-positive individuals (OR = 6.91; 95% CI 3.50–16.63). Specific diagnostic categories also showed large effect sizes, including obsessive–compulsive disorder (OR = Inf.; lower bound CI = 2.10) and other DSM-IV anxiety disorders (OR = 4.95; 95% CI 2.23–10.99). In contrast, among MDQ-positive individuals with high QoL, the effect sizes comparing psychiatric diagnoses to matched MDQ-negative controls were close to unity, indicating minimal differences consistent with an adaptive hyperactivation profile.

As shown in **Table 3**, the two MDQ-positive subgroups (high vs. low QoL) did not differ significantly in sex distribution or mean age. Owing to the matching procedure, the corresponding control groups displayed an identical demographic profile for these variables. No statistically significant differences emerged between cases and controls within each QoL stratum, confirming the adequacy of the matching process and the comparability of the four analytic subgroups.

**Table 3:** Characteristics of cases and controls by sex and age.

Cases	High QoL MDQ+ N=30	Low QoL MDQ- N=61	Statistics within cases and controls (MDQ vs L-MDQ+))	P
Sex (Females)	13	34	Chi-Square =1.239	0.372
Age	42.23±13.35	42.97±15.53	F=0.050 (1; 89 df)	0.824
Controls	High QoL MDQ+N=60	Low QoL MDQ-N=61	Statistics within cases and controls (MDQ vs L-MDQ+))	P
Sex (Females)	Matched 2/1 with MDQ+LowQoL+ 26	Matched 1/1 with MDQ-LowQoL 34	Chi-Square =1.872	0.172
Age	Matched with MDQ+QoL+ 42.23±13.35	Matched 1/1 with MDQ-LowQoL 42.97±15.53	F=0.079 (1; 89 df)	0.079

**Table 1** illustrates a clear differentiation within MDQ-positive individuals. Those with high QoL exhibit psychiatric comorbidity rates comparable to their MDQ-negative counterparts, supporting the hypothesis that a subset of MDQ-positive individuals reflects an adaptive hyperactivation phenotype rather than latent psychopathology. In contrast, MDQ-positive individuals with low QoL show a markedly higher burden of lifetime psychiatric diagnoses—including anxiety disorders, obsessive–compulsive disorder, and eating disorders—compared not only with the high-QoL MDQ-positive group but also with MDQ-negative controls with similarly low QoL. This dichotomy suggests that MDQ positivity encompasses both adaptive and dysregulated activation profiles, further highlighting the need for more refined screening tools capable of distinguishing functional hyperactivity from clinically significant dysregulation.

## Discussion

The study showed the existence of a minority of people positively screened by the MDQ who have high satisfaction with their quality of life and who present low frequencies of all psychiatric disorders, not dissimilar to that of people negative to the MDQ with an equally high perception of quality of life. The frequencies of psychiatric disorders in those people were lower than those of the others who were positive to the MDQ, without high satisfaction with the quality of life (73).

In revising the Discussion, we prioritized a clearer alignment between the empirical findings and the initial research questions. The results indicate that MDQ positivity captures a heterogeneous set of activation profiles, with a distinct subgroup exhibiting preserved functioning and minimal psychiatric morbidity—consistent with an adaptive hyperactivation phenotype. Conversely, MDQ-positive individuals with low QoL display elevated rates of anxiety, obsessive–compulsive symptoms, and eating disorders, suggesting a pattern of dysregulated activation. These contrasts directly address the study’s primary objective and underscore the need for diagnostic approaches capable of distinguishing functional from maladaptive hyperactivation. Speculative elements regarding potential biological or environmental mechanisms have been moderated and reframed as hypotheses to be examined in future longitudinal or mechanistic studies.

If we assume that among the individuals positive at MDQ with a high perception of quality of life, the (few) individuals with a previous lifetime psychiatric diagnosis are people with the condition in remission at the time of the interview, and so with a high score at SF-12, the low frequency of psychiatric diagnoses found in the group of MDQ positives becomes even more exceptional. It is, in fact, inconceivable that a possible recall bias concerns an episode of hypomania and no other more impairing psychiatric diagnosis. It is thus unlikely that individuals who remembered the episode of hypomania (resulting in a positive response to the MDQ) but, on the contrary, did not remember a previous period with phobia, Anxiety, or depression were recruited in the experimental group. On the contrary, a recall bias may have both interested hypomania and more depressive episodes and other psychiatric conditions (74). Still, it would have placed these individuals in the control group, contaminating the possibility of demonstrating the starting hypothesis and reinforcing the null hypothesis (therefore not invalidating the search results).

It seems, therefore, to confirm the hypothesis that the MDQ can screen different types of conditions as positive: people with a diagnosis of bipolar disorder but since the number of “false positives”, as this study also confirms, is excessive, the accuracy as a screener of MDQ is low; people with other psychiatric diagnoses without the bipolar disorder (with a high frequency of anxiety disorders and obsessive-compulsive disorders in this study); people without psychiatric diagnoses but with a low perception of quality of life and, as demonstrated by other studies with dysregulation of social rhythms and signs of stress (DYMERS syndrome); people with a good level of adaptation (in this case with a high perception of quality of life) therefore with previous episodes of hyperactivation/hyper-energy with adaptive meaning.

Recent research has highlighted how the current lifestyle that requires “accelerations” and sudden changes in social rhythms and underlying biological rhythms may have favored the adaptability of people with an aptitude for exploration and hyperactivity and the ability to “endure” periods of hyperactivity (67, 75). The aptitudes of exploration and search for novelty would expose these people both to possible success and, vice versa, to the fall into the dysregulation of rhythms and mental disorders if the stress became chronic (76).

It is, therefore, possible that modern lifestyles, just as they can increase the risk of bipolar disorders and of adjustment/traumatic disorders, may also produce a positive selection for people with basic traits of hyperactivity and novelty seeking and/or people capable of withstanding periods of substantial increases in activity with disruption of rhythms.

Light pollution typical of megalopolises in which people with hyperactivity traits have a possible well adjustment would also have modified the social context (77). Artificial light disrupts natural daily rhythms by enabling activities typically performed during daylight hours, such as eating or social interactions, to occur during periods of natural darkness, both indoors and outdoors. This disruption significantly affects the immune-endocrine circadian (24-hour) timing system and other endogenous rhythms, which have evolved to optimize human behavior by synchronizing it with natural light variations (for circadian rhythms) and other environmental factors like weather and seasonal changes (78). The desynchronization of biological rhythms caused by exposure to artificial light has been linked to metabolic dysfunction and obesity, with evidence supporting these associations (78, 79). Light pollution is, in fact, related to changes in the time stamp of melatonin secretion and, therefore, seems capable of modifying circadian rhythms and the secretion of sexual steroids toward a modification of the balance of activating steroids/stabilizing steroids to the advantage of the former Thus research has highlighted the potential implications for increased risks of breast and prostate cancers (80). However, this hormonal imbalance is also very likely a risk factor for bipolar disorder (70). Living in megalopolises with evident light pollution (as well as with a typically fast pace of life) is associated with a higher frequency of positive MDQ tests compared to people with the same genetic base but who remained in their areas of origin, free from light pollution and “accelerated life” (81). It is therefore very likely that the increased in frequency of people with MDQ+ without psychiatric disorders and well-adapted had risen even in that circumstance, in fact in the subsample of Buenos Aires where, in addition to the MDQ a screener of the depressive episode was conducted (which therefore included major depression and bipolar disorder). However, the higher frequency of MDQ positivity in the “Sardinians” of Buenos Aires, depressive disorders were much more frequent in the Sardinians resident in Sardinia than in those of Buenos Aires (82).

While the pattern observed among MDQ-positive individuals with high QoL is consistent with an adaptive hyperactivation phenotype, alternative explanations should also be considered. Preserved functioning may reflect individual resilience, stronger coping resources, or protective personality traits that enhance stress tolerance. Recent positive life events or periods of achievement may temporarily elevate perceived QoL despite the presence of activation symptoms. In addition, robust social support networks and reduced exposure to chronic stressors may further contribute to high functioning in this subgroup. These possibilities highlight that high QoL among MDQ-positive individuals may result from multiple interacting pathways, not solely from adaptive activation traits, and merit investigation in future longitudinal designs.

It should be noted that the considerations regarding genetic predisposition, circadian disruption, and environmental factors such as light pollution are speculative and extend beyond the empirical findings of the present study. These elements are discussed solely as hypothetical mechanisms that could inform future research on the interplay between activation traits, environmental pressures, and adaptation. While such hypotheses may offer useful theoretical perspectives, they should not be interpreted as conclusions supported by the current dataset, and require dedicated longitudinal and biological investigations.

The major limitation of this study is that the hypotheses on which it is based were born from reflections on the research results that started with other objectives and, therefore, with tools that were not adequate for the specific hypotheses. Given that the Mood Disorder Questionnaire can also select people with dysregulation of rhythms and hyperactivation syndrome (DYMERS) as well as people with adaptive hyperactivity (as the present research seems to confirm), however, this questionnaire is not adequate for the new hypotheses (in fact it screened all together those different conditions) thus it is necessary to think of new tools adequate to the new hypotheses.

The cross-sectional nature of this study prevents any inference about temporal or causal relationships between hyperactivation, quality of life, and psychiatric comorbidity. Reverse causality—whereby preserved functioning may shape the subjective report of activation, or vice versa—cannot be excluded. Accordingly, longitudinal cohorts are essential to clarify whether adaptive hyperactivation represents a stable trait, an early marker of resilience, or a transitional state preceding dysregulation. Future prospective designs will be crucial to map trajectories over time and to determine the prognostic significance of MDQ-positive profiles.

Beyond its cross-sectional nature, the case–control design inherently prevents causal inference. The associations observed between activation patterns, quality of life, and psychiatric morbidity cannot establish temporal precedence or directionality. Prospective longitudinal studies are needed to determine whether these profiles represent stable traits, transitional states, or early indicators of future clinical trajectories.

Because several diagnostic categories were compared across groups, the possibility of inflated Type I error must be acknowledged. We did not apply conservative corrections such as Bonferroni because the analyses were hypothesis-driven and focused on diagnostic clusters previously linked with MDQ positivity. Such corrections would have markedly increased the risk of Type II error, potentially masking clinically relevant associations in a population-based sample. Nonetheless, the results should be interpreted with appropriate caution, and future studies with larger samples may allow for more stringent adjustment procedures.

Although the sampling strategy ensured demographic comparability, the original survey did not include sufficiently detailed or standardized measures of socioeconomic status, educational attainment, or occupational category to permit formal adjustment. As these variables may influence both quality-of-life perception and the expression of activation traits, the possibility of residual confounding cannot be fully excluded. However, the population-based design and the matching on age and sex likely reduced major structural differences across groups.

Specifically, the MDQ has been developed to screen for “hyperactivation, “ encompassing pathological and potentially adaptive forms. However, the instrument was not intended to discriminate between the two, thus prompting the development of novel tools integrating the MDQ with measures of rhythm dysregulation, novelty seeking, and functioning, among other constructs. In addition, it is worth noting that while high SF-12 scores are herein assumed to point toward adaptive hyperactivation, additional factors such as socio-economic status, resilience, absence of recent stressors, beyond adaptive mood traits, may account for that. From this perspective, forthcoming longitudinal studies need to expand the factors putatively accounting for adaptive hyperactivation to rule out the chance of reverse causality of poor adaptation and high SF-12 scores.

These observations also confirm what emerged from our study, that is, that the MDQ can select (along with others) individuals with previous episodes of acceleration, low need for sleep, and hyperenergy but without psychiatric diagnoses. The study of the relationship of these pictures with mood pathology can be the objective of a future challenge of great interest.

### Clinical implications and integrative care suggestions

The present findings suggest that MDQ positivity should not be interpreted as a homogeneous marker of bipolar spectrum pathology. In clinical practice, the identification of individuals with preserved functioning and high quality of life—despite reporting lifetime hyperactivation—may help avoid over-pathologization and unnecessary initiation of mood stabilizers. Instead, these individuals may benefit from integrative, low-intensity interventions targeting stress reactivity and rhythm stability, including sleep–wake regulation, structured daily routines, psychoeducation on early warning signs, and lifestyle-based approaches that modulate social and biological rhythms. Conversely, MDQ-positive individuals with impaired quality of life should prompt clinicians to assess comorbid anxiety, obsessive–compulsive symptoms, and stress-related dysregulation. Viewing MDQ outcomes through this stratified clinical lens may support more precise diagnostic formulations and more proportionate, preventive, and person-centered care pathways.

## Conclusions

This study provides compelling evidence that a positive score on the MDQ identifies a heterogeneous population, encompassing not only individuals with bipolar spectrum disorders and psychiatric comorbidities but also a distinct subgroup characterized by adaptive hyperactivation, marked by preserved quality of life and minimal psychiatric morbidity. These findings challenge the traditional assumption that MDQ positivity invariably signals latent psychopathology and underscore the need to reconceptualize hyperactivation phenomena within a broader clinical and public health framework. The current results highlight the urgent need for refined diagnostic instruments to discern adaptive from maladaptive hyperactivity states, thereby avoiding over-pathologization and promoting more tailored preventive strategies. In light of contemporary societal dynamics favoring accelerated rhythms and heightened stimulation, the differentiation between pathological and adaptive activation may represent a crucial frontier for future psychiatric research, with profound implications for diagnosis, prognosis, and intervention in mood spectrum conditions.

## Data Availability

The raw data supporting the conclusions of this article will be made available by the authors, without undue reservation.
